# Tensile testing data of additive manufactured ASTM D638 standard specimens with embedded internal geometrical features

**DOI:** 10.1038/s41597-024-03369-y

**Published:** 2024-05-16

**Authors:** Youssef AbouelNour, Nick Rakauskas, Gabrielle Naquila, Nikhil Gupta

**Affiliations:** Composite Materials and Mechanics Laboratory, Mechanical and Aerospace Engineering Department, New York University, Tandon School of Engineering, 6 MetroTech Center, Brooklyn, NY 11201 USA

**Keywords:** Mechanical engineering, Mechanical properties, Composites

## Abstract

Additive manufacturing (AM) is now widely used for research and industrial production. The benchmark data for mechanical properties of additively manufactured specimens is very useful for many communities. This data article presents a tensile testing dataset of ASTM D638 size specimens without and with embedded internal geometrical features printed using polylactic acid (PLA) in a Fused Filament Fabrication (FFF) additive manufacturing process. The added features can mimic defects of various shapes and sizes. This work is a supplement to the published research article *Assisted defect detection by in-process monitoring of additive manufacturing using optical imaging and infrared thermography* (Additive Manufacturing, 2023, 103483). The printed specimens were tensile tested. Stress-strain graphs were developed and used to calculate the mechanical properties such as ultimate tensile strength (UTS) and strain at UTS. The mechanical properties, the correlations between mechanical properties and size, shape and location of geometrical features (defects), and the trends in mechanical properties can be useful in benchmarking the results of other researchers.

## Background & Summary

This data article is a supplement of a published research article^1^. Additive manufactured ASTM D638 standard specimens with embedded internal geometrical features were designed, printed using polylactic acid (PLA), and monitored by cameras in real-time for the purpose of non-destructive testing. The specimens were then tensile tested as a means of destructive testing. The data presented in the article provide mechanical properties of the specimens that were obtained via tensile testing, such as ultimate tensile strength (UTS) and strain at UTS. The objective of this data article is to offer researchers, along with the published research article, a comprehensive overview of the mechanical characteristics of specimens printed with and without embedded internal features. Some of these features may represent defect of different types and sizes, providing a benchmark for researchers observing naturally occurring defects in their printed parts to estimate the trends in the mechanical properties of the parts having lower or higher number of defects and of different sizes.

ASTM D638 size specimens with several variations of internal features, which were created to mimic various defect shapes and patterns, were designed in SolidWorks and import into Ultimaker Cura 4.9.1 for slicing. Internal features were randomly embedded in the specimens. The specimens were then additively manufactured using a Prusa i3 MK3S+ 3D Printer. Printed specimens were tensile tested using an Instron 4400 Series Universal Testing Machine with a 50 kN load cell. Images of the fractured specimens were acquired using an iPhone 11 camera. Raw data was exported from the machine into.csv format. This data was organized into multiple sheets based on trials and specimen types.

Internal features were seeded by altering the extrusion pattern to create voids and delay extrusion commands while keeping the specimen weight unchanged or by reducing the weight of the specimens. Generated G-code files with embedded internal features, or specimen *Types*, were named alphabetically, ranging from *Type A* to *Type L*. In these files, the G-code modification takes place every α number of layers, where α corresponds to 20, 20, 8, 5, 4, and 3 layers for specimen types *A* to *L*, respectively. Hence, the total number of defects in specimen types *A* to *L* were 146, 297, 369, 593, 736, and 982, respectively. The defects were not seeded in the first and last 25% of the layers to ensure only invisible internal defects were embedded in the specimens. Please see^1^ and^2^ for more details on generation of the G-code files and specimen *Types*. Specimen dimensions were measured and input into the Instron machine prior to tensile testing. Tensile testing results were used to support the conclusions presented in^3^. A one-inch extensometer was mounted on the specimens, which were gripped in the machine using a wedge action tensile grip. Load and displacement values were obtained for each specimen from the machine and were converted to stress and strain values using the specimen cross section area and gauge length, respectively. These stress-strain curves were used to calculate UTS and strain at UTS.

## Methods

A standard ASTM D638 specimen was designed in SolidWorks and printed using fused filament fabrication (FFF) based Prusa i3 MK3S+ 3D Printer using PLA material. Several batches of the standard defect-free specimen were printed to enable repeat testing and statistical analysis of results data.

**Fig. 1:**
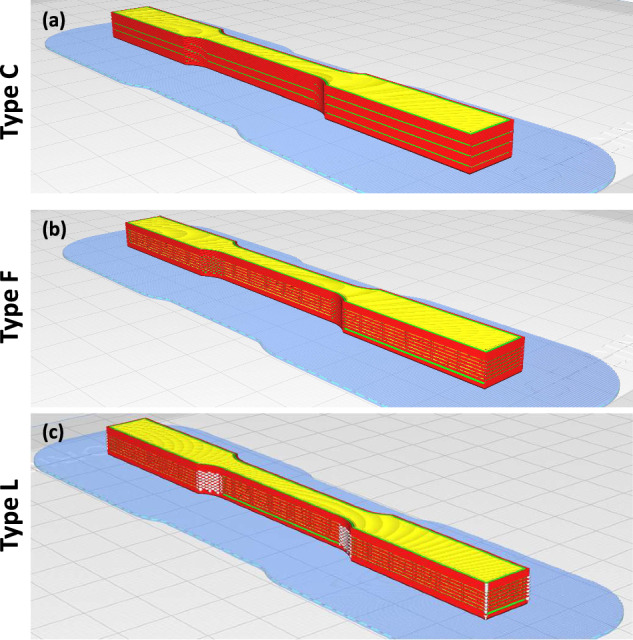
Isometric views of ASTM D638 specimen *Types C, F*, and *L* in Cure showing an increasing number of embedded defects in the specimens in ascending order alphabetically.

The internal features were embedded by alteration of the standard specimen’s g-code. The output was 17 g-code files, which were named *Types A* through *Q*. The specimen *Types A* through *L* were printed 7 times each and *Types M* through *Q* were printed 3 times each. See Fig. 1 for isometric views of representative specimen *Types C, F*, and *L* in Cura, showing the different quantities and variations of embedded defects in the specimens.

Specimens *Types A* through *Q* varied depending on the number of embedded point and line features in the specimens or material quantity. For *Types A* through *F*, extrusion patterns were delayed every *x* lines of G-code and this material was deposited at the end location before the next movement. For *Types G* through *L*, extrusion patterns were delayed every *x* lines of G-code and this material was deposited in excess at the next extrusion. For *Types A* through *F* and *Types G* through *L, x* was given the value of 20, 10, 8, 5, 4, and 3, respectively. This implies that the number of defects increased as the *x* value decreased.

**Fig. 2:**
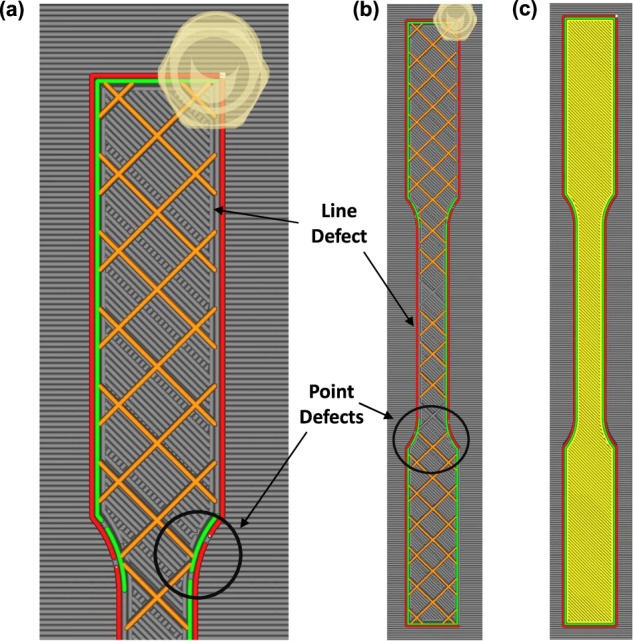
ASTM D638 specimens in Cura. (**a**) Sectional view showing point and line defects, (**b**) complete extruder movement for the formation of the specimen with embedded defects, and (**c**) completed specimen.

Due to the raster pattern and defect’ locations in the G-code, two types of defects were formed in these specimens, classified as point and line defects^1^. Point defects are ~1 mm in length, while line defects ≥ 30 mm in length. The frequency of each of these defects was dependent on the delayed extrusions and *x*. Therefore, the position of a defect within a particular layer, as well as infill density were directly correlated to G-code alternation, and hence, *x*. See Fig. 2 for sectional and complete views of extruder movement in Cura and the different types of defects that were embedded in the specimens (i.e., point and line defects).

For *Types M* through *Q*, ASTM specimens were printed with 90, 80, 70, 60 and 50% material reduction, respectively.

**Fig. 3:**
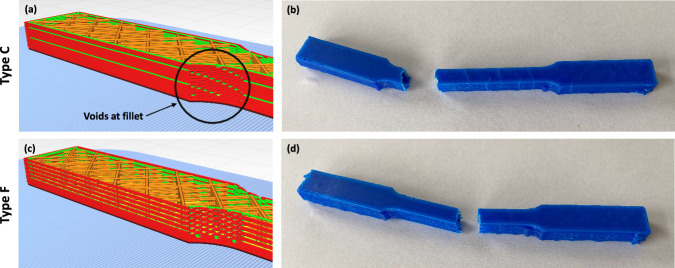
(**a,c**) Sectional views of specimen *Types C* and *F* in Cura, respectively, and (**b,d**) images of the corresponding fractured specimens after tensile testing.

All specimens were then tensile tested. Specimens had a nominal length of *L* = 111 mm and cross-sectional area of *A* = 60 mm². For each tensile test, an extensometer was installed onto the specimen to measure extension (mm), which was then divided by the specimen length to acquire *strain* values (mm/mm). For each tensile test, the machine was calibrated. The testing was conducted until the specimens fractured. Stress (MPa) values were obtained by diving the load by *A*. See Fig. 3 for sectional views of specimen *Types C* and *F* in Cura and images of the corresponding fractured specimens after tensile testing.

Following tensile testing, raw data was extracted to present only relevant tensile testing data: from loading of the specimen to fracture. Stress-strain graphs were plotted on the exported Excel sheets for future evaluation of each specimen’s mechanical properties. The UTS (MPa) and strain at UTS (mm/mm) were obtained for each specimen. These values are recorded in the data files. Tensile testing results for specimen *Types A* through *L* and the *No Defects* specimen are presented in the following files included in the data repository^4^:

“Trial 1 – A through L.xlsx”,

“Trial 2 – A through L.xlsx”,

“Trial 3 – A through L.xlsx”,

“Trial 4 – A through L.xlsx”,

“Trial 5 – A through L.xlsx”,

“Trial 6 – A through L.xlsx”,

“Trial 7 – A through L.xlsx”,

“Trial 8 – M through Q.xlsx”,

“Trial 9 – M through Q.xlsx”,

“Trial 10 – M through Q.xlsx”, and

“No Defects.xlsx”.

Images of the fractured specimen types were captured using an iPhone 11 Camera. Images are included in the compressed folder named “Images of Fractured Specimens.zip”.

## Data Records

The dataset consists of 11.csv files with multiple sheets and a compressed (.zip) folder. Each sheet in a .csv file consists of five columns of data labelled *time, extension, load, tensile strain*, and *stress*; the length, *L*, and cross-sectional area, *A*, of the specimen; a stress-strain graph; the ultimate tensile strength (UTS) of the specimen; and the strain at UTS. The compressed (.zip) folder provides images of the fractured specimens. A summary of the dataset is provided in Table 1.

**Table 1:** Dataset specifications.

File Name	Number of Sheets	Sheet Names
No Defects.xlsx	10	*“0_1”* through *“0_10”*
Trial 1 - A through L.xlsx	12	*“A”* through *“L”*
Trial 2 - A through L.xlsx	12	*“A”* through *“L”*
Trial 3 - A through L.xlsx	12	*“A”* through *“L”*
Trial 4 - A through L.xlsx	12	*“A”* through *“L”*
Trial 5 - A through L.xlsx	12	*“A”* through *“L”*
Trial 6 - A through L.xlsx	12	*“A”* through *“L”*
Trial 7 - A through L.xlsx	12	*“A”* through *“L”*
Trial 8 - M through Q.xlsx	5	*“M”* through *“Q”*
Trial 9 - M through Q.xlsx	5	*“M”* through *“Q”*
Trial 10 - M through Q.xlsx	5	*“M”* through *“Q”*
Images of Fractured Specimens.zip	—	—

The dataset is provided in the Mendeley Data repository, see^4^.

## Technical Validation

The ASTM standard geometries of the specimens were additively manufactured using PLA material. A batch of these specimens was pristine, without any internal features, to provide the baseline properties. The tensile test results obtained from these specimens were used for validation of the test results obtained from the specimens that contained internal features. In addition, in order to develop statistical analysis, at least five specimens of the pristine designs were printed and tested. The calculation of standard deviation was conducted to ensure that they were within the generally accepted ±5% of the mean values. The presence of internal design features, which act as defects in the specimens, decrease the strength of the specimens as the number of defects in the gauge section of the specimens increases. These trends are represented through stress-strain graphs that were created using tensile testing results^5,6^.

**Fig. 4:**
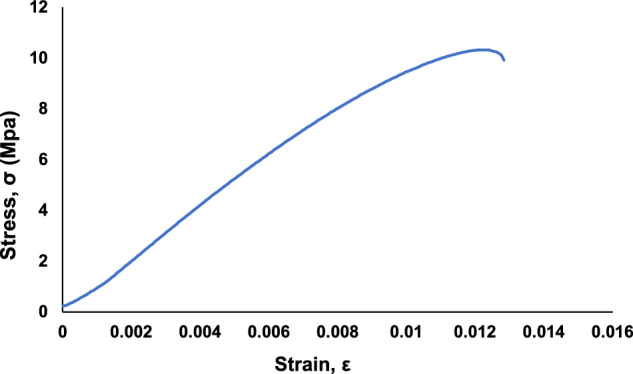
Stress-strain graph for tensile testing results of a representative pristine specimen.

**Fig. 5:**
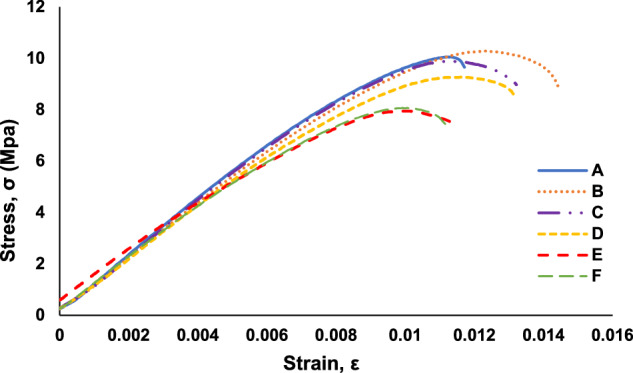
A representative set of stress-strain graphs for tensile testing results of specimen *Types A* through *F* (Trial 5).

**Fig. 6:**
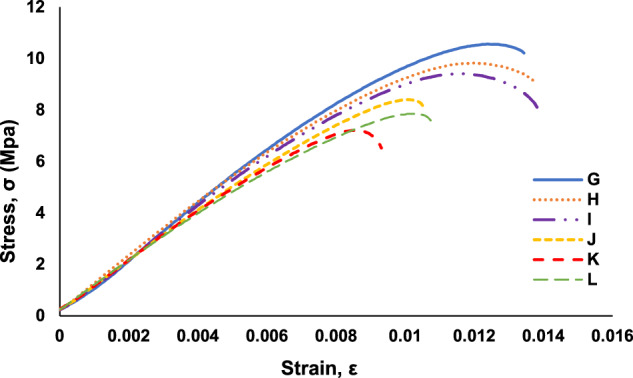
A representative set of stress-strain graphs for tensile testing results of specimen *Types G* through *L* (Trial 5).

**Fig. 7:**
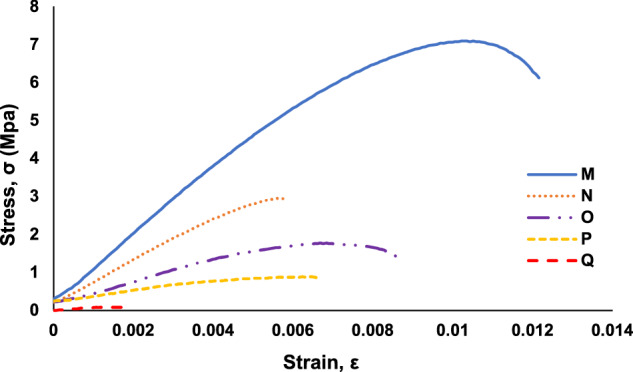
A representative set of stress-strain graphs for tensile testing results of specimen *Types M* through *Q* (Trial 8).

The acquired load-displacement data was converted to stress-strain graphs by using the specimen dimensions and gauge length. The stress-strain graphs are included in the data repository files and are used for calculation of the ultimate tensile strength (UTS) and strain at UTS, which are used to understand trends in the datasets related to a decrease in mechanical strength with an increase in embedded defects. A stress-strain graph for a pristine specimen is shown in Fig. 4. As samples, stress-strain graphs for each specimen in “Trail 5 – A through F” are shown in Fig. 5. Stress-strain graphs for each specimen in “Trail 5 – G through L” are shown in Fig. 6. Stress-strain graphs for each specimen in “Trial 8 – M through Q” are shown in Fig. 7.

**Fig. 8:**
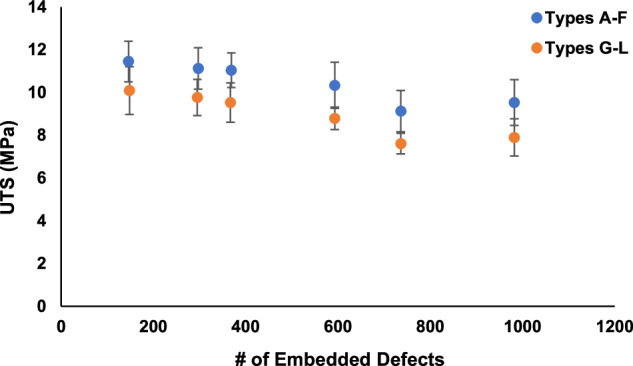
Tensile testing results of ASTM D638 specimens *Types A* through *L*. UTS vs. the number of embedded defects in the specimens and (b) strain at UTS vs. the number of embedded defects in the specimens^3^.

**Fig. 9:**
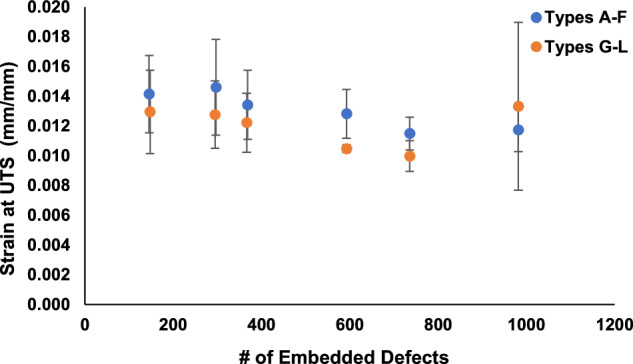
Tensile testing results of ASTM D638 specimens *Types A* through *L*. Strain at UTS vs. the number of embedded defects in the specimens^3^.

Furthermore, Figs. 8, 9 provide comparisons of the average values of UTS and strain at UTS vs. the average number of embedded features in the specimens, respectively. As shown in Fig. 8, UTS decreased with standard errors of ~9% for *Types A* through *F* and *Types G* through *L* as the number of internal design features in the specimens increased^3^. As shown in Fig. 9, for strain at UTS, there was similar trend to that shown for UTS, but with larger standard errors.

## Data Availability

There was no code used for the generation of the datasets.
